# Increase in tumour permeability following TGF-*β* type I receptor-inhibitor treatment observed by dynamic contrast-enhanced MRI

**DOI:** 10.1038/sj.bjc.6605367

**Published:** 2009-11-03

**Authors:** T Minowa, K Kawano, H Kuribayashi, K Shiraishi, T Sugino, Y Hattori, M Yokoyama, Y Maitani

**Affiliations:** 1 Institute of Medicinal Chemistry, Hoshi University, Ebara 2-4-41, Shinagawa, Tokyo 142-8501, Japan; 2 Varian Technologies Japan Limited, Shibaura 4-16-36, Minato, Tokyo 108-0023, Japan; 3 Kanagawa Academy of Science and Technology, Sakado 3-2-1, Takatsu, Kawasaki, Kanagawa 213-0012, Japan; 4 Department of Basic Pathology, Fukushima Medical University, Hikariga-oka 1, Fukushima 960-1295, Japan

**Keywords:** MRI, liposome, angiogenesis, TGF-*β* inhibitor, contrast agent, tumour

## Abstract

**Background:**

To enhance the success rate of nanocarrier-mediated chemotherapy combined with an anti-angiogenic agent, it is crucial to identify parameters for tumour vasculature that can predict a response to the treatment of the anti-angiogenic agent.

**Methods:**

To apply transforming growth factor (TGF)-*β* type I receptor (T*β*R-I) inhibitor, A-83-01, to combined therapy, dynamic contrast-enhanced magnetic resonance imaging (DCE-MRI) was carried out in mice bearing colon 26 cells using gadolinium (Gd)-DTPA and for its liposomal formulation to evaluate changes in tumour microvasculature following A-83-01. Tumour vascular parameters from DCE-MRI were compared with histological assessment and *apparent diffusion coefficient* of water in tumour generated by diffusion-weighted MRI.

**Results:**

Contrary to evaluations reported for anti-angiogenic agents, A-83-01 treatment increased the initial area under the Gd concentration–time curve (IAUGC_60_), volume transfer constant (*K*^trans^) and fractional plasma volume (*v*_p_) significantly within 24 h, that was positively related to *α*-smooth muscle actin-positive pericyte coverage and tumour cell proliferation, and was correlated inversely with the *apparent diffusion coefficient*. The vascular function of the tumour improved by A-83-01 treatment was well assessed on post-liposomal Gd-DTPA-enhanced MR images, which predicted delivery of a liposomal drug to the tumour.

**Conclusion:**

These findings suggest that DCE-MRI and, in particular, *K*^trans^ and *v*_p_ quantitation, provide important additional information about tumour vasculature by A-83-01 treatment.

The success of chemotherapeutic agents with solid tumours is critically dependant on the access that these agents have to the tumours via the so-called ‘leaky vasculature’. In particular, tumour vasculature is crucial for the delivery of drugs encapsulated in nanocarriers (Matsumura and Maeda, 1986). Anti-angiogenesis effects are known to change the tumour vasculature; therefore, this technique has been already applied to combined therapy. Bevacizumab, an anti-vascular endothelial growth factor (VEGF) antibody, was developed for blocking angiogenesis and it is clinically used with other drugs to improve the efficiency of chemotherapy.

The roles of transforming growth factor (TGF)-*β* in cancer biology are complex; TGF-*β* can suppress or promote tumour growth depending on the type of cancer. Small molecule TGF-*β* type I receptor (T*β*R-I) inhibitor has a wide variety of effects (Jakowlew, 2006; Tsuchida *et al*, 2006). The T*β*R-I inhibitor LY364947 was reported to increase the accumulation of an anti-cancer drug encapsulated in nanocarriers by changing the micro-environmental vasculature (Kano *et al*, 2007). The T*β*R-I inhibitor A-83-01 is one of more potent inhibitors of T*β*R-I kinase/activin receptor-like kinase (ALK)-5 (IC_50_=12 nM) (Tojo *et al*, 2005) than a previously described ALK-5 inhibitors including LY364947 (IC_50_=59 nM) (Li *et al*, 2006), although the *in vivo* effect has not been made known. To estimate the tumour state after treatment with T*β*R-I inhibitor is important to determine an administration schedule for T*β*R-I inhibitor-combined therapy. However, it is difficult to rationally determine whether tumour blood vessels are amenable to nanocarrier-mediated therapy in an individualised manner.

Dynamic contrast-enhanced magnetic resonance imaging (DCE-MRI) is one of the evaluation methods of anti-angiogenic agents, such as anti-VEGF antibody and tyrosine kinase inhibitor, clinically (Morgan *et al*, 2003; O’Connor *et al*, 2007) and preclinically (Marzola *et al*, 2004; Nakamura *et al*, 2006; Bradley *et al*, 2009), by calculating pharmacokinetic parameters, including fractional plasma volume (*v*_p_) and the volume transfer constant (*K*^trans^) from the enhancement of tumour signal intensity by gadolinium (Gd) contrast agent (Tozer, 2003; Kiessling *et al*, 2007). To my knowledge, however, there are no reports to evaluate T*β*R-I inhibitor by DCE-MRI. In clinical studies, small molecular weight contrast agents, Gd chelates, have been used. *K*^trans^, the Gd exchange constant between blood and tumour interstitial tissue, depends on the balance between permeability and blood flow. Therefore, the *K*^trans^ parameter depends on the size of the contrast agent. The choice of the optimal contrast agent is considered to be essential for a successful characterisation of tumour angiogenesis. As macromolecule contrast media show lower permeability than Gd cheleates, it is useful for permeability change monitoring in tumour vasculature (Daldrup-Link *et al*, 2004; Turetschek *et al*, 2004); Liposomes are self-closed colloidal particles in which bilayer membranes composed from self-aggregated lipid molecules encapsulate a fraction of the medium. Liposomes have been used as drug carriers for anticancer drugs such as Doxil. For this reason, liposomal Gd has a substantial potential to detect permeability-limited conditions. There are still no reports on the use of liposomes as a DCE-MRI contrast agent. Furthermore, liposomal contrast agents to evaluate nanocarrier behaviour in tumour directly will be a hopeful method of examination for combination therapy.

Thus, the purpose of this study was to evaluate changes in tumour vasculature as parameters using DCE-MRI to monitor responses in mice following A-83-01 administration. In addition to DCE-MRI, diffusion-weighted imaging was used to estimate *the apparent diffusion coefficient* of tissue water (Koh and Padhani, 2006; Patterson *et al*, 2008). T*β*R-I inhibitor activity was also evaluated in representative experiments through tumour vascularity, the proportion of endothelial cells associated with pericytes, and microvessel density from histological slices.

## Materials and methods

### Animals

All animal experiments were carried out in accordance with the guidelines of the Guiding Principles for the Care and Use of Laboratory Animals of Hoshi University. Colon 26 cells (1 × 10^6^) were inoculated subcutaneously into the right back at the side of the heart in CDF1 female mice (6-weeks old, Sankyo Labo Service, Tokyo, Japan). When the tumour size reached approximately 100 mm^3^, A-83-01 (Sigma Chemical, St. Louis, MO, USA) (Supplementary Figure S1A) dissolved in DMSO/saline=3 out of 2 (v/v) was injected intraperitoneally. The tail vein was catheterised post-injection of contrast agent during the DCE-MRI experiment. Mice were anaesthetised with 1.5% isoflurane (Abbott Japan, Tokyo, Japan) throughout the MRI experiment during their insertion into a 9.4T vertical type MRI (Varian, Palo Alto, CA, USA). For a single treatment of A-83-01, mice (N=4) were injected with A-83-01 at a dose of 1 mg kg^−1^ at ‘0 h’ (Supplementary Figure S1B). In this experiment, 0 h was the time of the first A-83-01 intraperitoneal injection and the number of hours represents time after the first injection of A-83-01. For repeated treatment, mice (N=4) were injected with A-83-01 at 0 and 21 h at the same dose as for the single treatment.

### Preparation of liposomal Gd-DTPA

For the preparation of liposomal Gd-DTPA (Gd-L), mixture of egg phosphatidylcholine (Q.P. Company, Tokyo, Japan), cholesterol (Wako Pure Chemical Industries, Osaka, Japan), and polyethyleneglycol 2000-distearoyl phosphatidylethanolamine (NOF, Tokyo, Japan) in a molar ratio of 5 : 2 : 0.35 was dissolved in ethanol at 60°C, hydrated with Gd-DTPA (Magnevist, Bayer-Schering Pharma AG, Berlin, Germany), stirred, and evaporated under a vacuum to remove ethanol. This mixture was exposed to ultrasound until the particle diameter was about 120 nm, followed by exhausted dialysis against phosphate buffered saline (pH 7.4) solution. The particle size of the liposomes was determined at 25°C using an ELS-Z2 instrument (Otsuka Electronics, Tokyo, Japan). The Gd concentration was determined using *inductively coupled plasma* with an SPS7800 apparatus (SII NanoTechnology, Tokyo, Japan). T^1^ relaxation times of Gd-L and Gd-DTPA were measured over the concentration range of 0–0.25 mM Gd at 9.4 T_1_ at room temperature. Relaxivity (R^1^) was then determined from the slope of the linear regression fits of 1/T_1_ *vs* the Gd concentration: 1/T_1_=R_1_ × [Gd]+1/T_10_, where T_10_ represents T_1_ of 0 mM Gd solution. R_1_ of Gd-L was 4.48 mM^−1^s^−1^, which was similar to that of Gd-DTPA (4.39 mM^−1^s^−1^).

### MRI

*Apparent diffusion coefficient* was estimated and mapped from *diffusion-weighted imaging* using the following parameters: repetition time (TR)=2000 ms, echo time (TE)=45 ms, slice thickness 3 mm, 64 × 64 data matrix, axial orientation, and field-of-view of 3 × 3 cm^2^. Three slices through the centre of the tumour were acquired. Diffusion gradients equivalent to b-values of 0, 200, 400, and 800 s mm^−2^ were employed using gradient pulse widths of *δ*=7 ms and Δ=20 ms.

Dynamic contrast-enhanced magnetic resonance imaging was carried out with Gd-DTPA and Gd-L before (‘pre’) and after treatment in each animal. With the use of Gd Gd-L, injected lipids containing Gd-L were retained in the tumour; therefore, different mice were used to compare pretreatment with treatment of A-83-01. Before DCE-MRI high spatial resolution, two-dimensional T_2_-weighted spin–echo axial images were acquired to detect the tumour position. Pre-contrast tumour T_1_ was determined using an inversion recovery-prepared spoiled gradient-recalled echo (SPGR) sequence. The inversion-recovery was carried out using a 180° hard RF pulse followed by a gradient crusher pulse. Inversion times were 0.2, 0.4, 0.8, 1.4, 2, and 3 s. The other MRI parameters were: TE=3 ms, field-of-view=3 × 3 cm^2^, slice thickness=4 mm, and matrix size=64 × 64. Both RF and gradient spoilers were applied. In DCE-MRI acquisition, it was applied repeatedly to acquire the axial slice SPGR images through the tumour and left ventricle with a second temporal resolution over 6 min: TR=7.8125 ms, TE=2.06 ms, matrix resolution=64 × 64, field-of-view=3 × 3 cm^2^, slice thickness=4 mm, flip angle=30°, number of slices=1, and two signal averages. Approximately 20 s of baseline DCE-MRI images were acquired. Gd-DTPA or Gd-L was administered at 20 *μ*l g^−1^ (0.1 mmol Gd kg^−1^) as a bolus with heparinized saline (total volume ∼0.4 ml) via manual injection over 2–3 s.

### Quantitative evaluation of MRI

Tumour regions-of-interest (ROI) covering the whole tumour was segmented on the T_2_-weighted axial images, using ImageJ software (NIH, Bethesda, MD, USA), and the tumour ROI was transferred to the *apparent diffusion coefficient* map calculated from *diffusion-weighted imagings*.

A T_1_ map of tumour was prepared by the imaging of the inversion-recovery method to quantitate tumour Gd concentrations. The concentration of Gd at each imaging time point in each voxel was estimated using the formula Bradley *et al* (2009) used. T_1_ in blood plasma at 9.4 T was 2.2 s, as reported previously (Tsekos *et al*, 1998) and T_1_ in tumour was from the T_1_ map. 1/T_1_ (t) was calculated for every time point for the blood and tumour Gd concentrations . The initial area under the Gd concentration–time curve over 60 s (IAUGC_60_) was calculated. The tumour haemodynamic parameters *K*^trans^ and *v*_p_ were calculated using a two-compartment model (Ewing *et al*, 2003). The plasma concentration over time was calculated from the left ventricle data, which were averaged for all mice in the Gd-DTPA and Gd-L groups for this value.

### Histological and immunohistochemical analysis

For the histological assessment of A-83-01 effects on tumour vasculature, tumour sections were observed at 24 h after repeated injection of A-83-01. Each of four tumours from A-83-01 treated and untreated mice was resected and fixed with 10% formalin. Paraffin-embedded samples were sliced into 3 *μ*M sections for *hematoxylin and eosin* staining and immunostaining. Antibodies against *α*-smooth muscle actin (SMA) (DAKO, Glostrup, Denmark) were used to identify the pericyte and anti-CD31 (Abcam, MA, USA, USA), endothelial cell marker and anti-Ki67 (Labvision, Fremont, CA, USA) antibodies to recognise the growth state cells (G1, S, and M phase). Vascular areas within the tumours were measured as the index of tumour vascularity by stained with anti-CD31. Five fields of tumour sections were analysed at low magnification using a computerised image analyser (Image-Pro Plus, Media Cybernetics, MA, USA). The ratio of vessel area against tumour area without necrosis was calculated.

### Statistical analysis

Values were expressed as the mean±s.d. A two-tailed Student's *t*-test was used comparison between the pre- and post-treatment groups. ANOVA analysis, followed by Dunnett's test, was used for multi-group comparisons. Pearson's correlation coefficients were used for determination between a significant positive and negative relationship. Correlations between 0.4 and 0.6 were considered moderate, whereas correlations from 0.7 to 1.0 were considered strong. Significant differences were accepted when the *P*-value was below 0.05.

## Results

**Figure 1:**
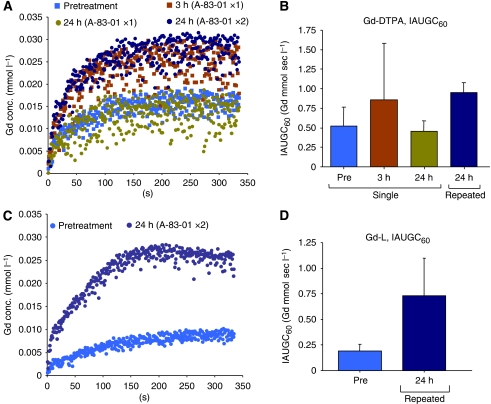
Mean gadolinium (Gd) uptake curves from regions-of-interest (ROI) over the whole tumour before (pre) and at different time points after (post) intraperitoneal A-83-01 injection with Gd-DTPA (**A**) and Gd-L (**C**), and IAUGC60_0−60_ with Gd-DTPA (**B**) and Gd-L (**D**). Data points (**B**, **D**) indicate mean±s.d. (N=3–6).

Figure 1A shows change of the Gd concentrations in DCE-MRI acquisition using Gd-DTPA at pretreatment, at 3-h, and 24-h post-injection of A-83-01. A progressive accumulation of Gd in the tumour was observed during the first 60 s followed by a plateau phase. The group treated with a single injection of A-83-01 showed the highest accumulation at 3 h post-injection of A-83-01 (1.7-fold the IAUGC_60_, Figure 1B) associated with a larger s.d., and a similar level to those with pretreatment at 24 h (0.9-fold the IAUGC_60_). At 24 h after repeated injection, the tumour accumulation increased a similar level to that at 3 h after the single injection (1.8-fold the IAUGC_60_). Next, we observed changes of the tumour vasculature repeat-treated by A-83-01 using Gd-L. The Gd concentration in DCE-MRI acquisition of untreated mice was very low (Figure 1C). The Gd concentration with Gd-L in repeat-treated mice increased during the first 200 s, and reached the same plateau value as with that of the Gd-DTPA repeat-treated mice. Eventually the repeated A-83-01 treatment increased 3.8-fold the IAUGC_60_ of Gd-L (Figure 1D), indicating a dramatic improvement in liposomal contrast agent delivery to the tumour.

**Figure 2:**
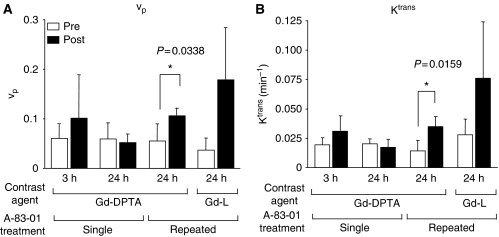
Values of fractional plasma volume (*v*_p_) (**A**) and volume transfer constant (*K*^trans^) (**B**) in the tumour before (pre) and at 3 h and 24 h after (post) single or repeated A-83-01 injections using gadolinium (Gd)-DTPA and Gd-L as a contrast agent. Each column represents the mean±SD (N=3 to 6).

From the data obtained above, *v*_p_ and *K*^trans^ values were calculated (Figure 2). With Gd-DTPA at 3 h after the single treatment, *v*_p_ and *K*^trans^ were high values accompanied with great variability, whereas at 24 h, *v*_p_ and *K*^trans^ were similar to those of the respective pretreatment values, suggesting that A-83-01 induced a transient change in the vasculature at around 3 h. On the other hand, at 24 h after repeated treatment, all mice showed increased *v*_p_ and *K*^trans^ with Gd-DTPA (*P*<0.05) and Gd-L. At 3 h after the first treatment, *v*_p_ and *K*^trans^ did not show significantly elevated values with Gd-DTPA, therefore, it can be concluded that the repeated administration schedule changed the tumour state positively for better liposomal contrast-agent distribution. The most characteristic point of the *v*_p_ and *K*^trans^ changes was the large dispersion of *v*_p_ and *K*^trans^ values after repeated A-83-01 treatments with the use of Gd-L. The diversity of local permeability of treated tumours may lead to large dispersion of Gd-L. The mouse tumour core showed an increase in the Gd concentration, as shown in Figure 1C, whereas the tumour rim showed a high peak concentration at about 1 min post-injection of Gd-L that then decayed (data not shown). In contrast, Gd-DTPA increased tumour Gd concentration homogeneously. This finding suggests that Gd-L could detect small changes in tumour micro-environments and brought about a big dispersion of *v*_p_ and *K*^trans^ values among treated mice.

**Figure 3:**
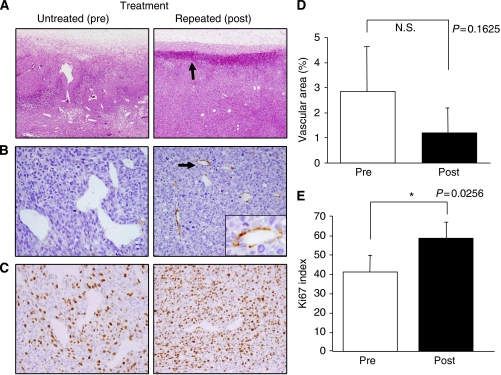
Histological analysis of tumours in untreated (pre) and treated (post) mice 24 h after repeated A-83-01 treatment. (**A**) *Hematoxylin and eosin* staining ( × 40). Arrow indicates that zonal bleeding was observed at the periphery of the tumour with A-83-01 treatment. (**B**) Immunostaining with anti-smooth muscle actin (SMA) antibody ( × 200 and inset, × 400). Irregularly dilated tumour vessels in untreated mice are not associated with pericytes, whereas the normalized vessels after A-83-01 treatment are surrounded by SMA-positive pericytes (arrow). (**C**) Immunostaining with Ki67. Ki67-positive proliferating tumour cells in the perivascular region are more prominent in the A-83-01 treated tumour than the untreated tumour ( × 200). (**D**) Mean percentage of the vascular areas within the tumours as the index of tumour vascularity. (**E**) Ki67 index in perivascular regions of (**C**). Proliferating tumour cells were increased significantly in A-83-01 treated tumours compared with untreated tumours (*P*<0.05). Each column represents the mean±s.d. (N=5).

Figure 3 shows histological observations of the tumours with or without the repeated A-83-01 treatment. Two distinct changes were observed, although there was no difference in tumour cell shape or necrosis. The first was intra-tumoural bleeding, which was exclusively configured at the periphery of the A-83-01-treated tumours with 200–300 *μ*m width and 100 *μ*m depth (Figure 3A). The bleeding lesions were not accompanied with tissue oedema, suggesting minute rupture of tumour vessels. This means that hyper-permeability had not occurred. The localised bleeding state may correspond to the accumulation site of Gd-L. The second observation was morphological changes of the tumour vasculature. Abnormal blood vessels with irregular dilation were seen in the untreated tumours, whereas the vasculature in A-83-01-treated mice was smaller, and its shape was more round, suggesting the vascular normalisation (Figure 3A). Tumour vascularity, the percentage of vascular area (1.2%) in the treated tumours (post) was not significantly lower than in the untreated tumours (pre, 2.9%, *P*>0.05), as the change was very diverse within a tumour (Figure 3D). The abnormal tumour vessels were not accompanied with pericytes, which were identified because of SMA reactivity (Figure 3B). It is interesting to note that the normalised vessels in tumours treated with A-83-01 were surrounded by pericytes (Figure 3B). The Ki67 index (58.5%) was significantly higher in the perivascular region of the A-83-01-treated tumours compared with the untreated tumours (41.4%, *P*<0.05) (Figure 3C, E). These findings suggested that the repeated A-83-01 treatment allowed the recovery of blood flow during 24 h.

**Figure 4:**
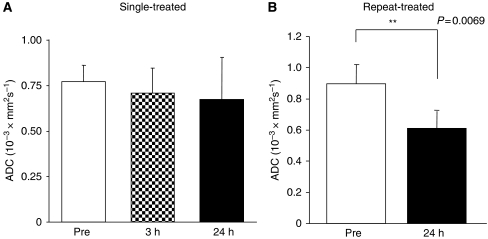
*Apparent diffusion coefficient* of the tumours before (pre) and at different time points after (post) single (**A**) and repeated (**B**) intraperitoneal A-83-01 injection. Repeat-treated tumours showed significant decreases in *apparent diffusion coefficient* compared with pretreatment. Each column represents the mean±s.d. (N=4).

In the evaluation of *apparent diffusion coefficient* value, single-treated groups at 3 and 24 h did not show a difference compared with the pretreatment, but the repeat-treated group at 24 h showed a significant difference (*P*<0.01, Figure 4). Alteration of extra- and intracellular fluid volume balance in repeat-administrated protocol may occur in the tumour because the diffusion rate of intracellular water is 1 order of magnitude smaller than that of the extracellular water (Van Zijl *et al*, 1991; Gass *et al*, 2001).

**Figure 5:**
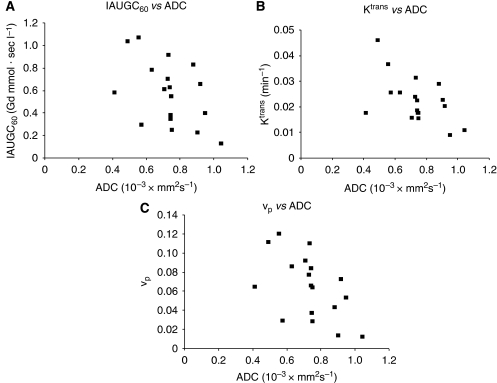
IAUGC_60_, transfer constant volume transfer constant (*K*^trans^), and fractional plasma volume (*v*_p_) with gadolinium (Gd)-DTPA *vs* tumour *apparent diffusion coefficient* (*ADC*). There was a moderately negative correlation between the IAUGC_60_ and ADC (r=–0.4774, *P*=0.0451, N=18) (**A**), between *K*^trans^ and ADC (r=–0.5333, *P*=0.0227, N=18) (**B**), and between *v*_p_ and ADC (r=−0.5253, *P*=0.0252, N=18) (**C**).

Next, the relation of DCE-MRI parameters with Gd-DTPA to tumour *apparent diffusion coefficient* was investigated (Figure 5). There was a moderately negative correlation between *the IAUGC*_60_ (Figure 5A),*K*^trans^(Figure 5B),*v*_p_, *and apparent diffusion coefficient* (Figure 5C). This suggests that these parameters may be of value in the assessment of tumour behaviour.

## Discussion

In this study, effects of a T*β*R-I inhibitor was firstly evaluated by means of DCE-MRI with Gd-DTPA and Gd-L in mice bearing colon 26 tumours. The effect of A-83-01 exhibited high IAUGC_60_, *v*_p_, and *K*^trans^ values at 24 h after repeated treatment.

An increase in *K*^trans^ by the use of Gd-L could conceivably increase the permeability and surface area of the capillary endothelium. The *K*^trans^ value estimated with Gd-L (*K*^trans^=0.076±0.048 min^−1^) after the repeated A-83-01 treatment was higher to that with Gd-DTPA (*K*^trans^=0.035±0.009 min^−1^) (Figure 2B). Liposomal contrast agents are promising for characterising the tumour vascularity and the angiogenesis status through DCE-MRI method.

Anti-angiogenic agents such as anti-VEGF antibody and tyrosine kinase inhibitor were reported that decrease both *K*^trans^ and IAUGC (O’Connor *et al*, 2007; Bradley *et al*, 2008; Bradley *et al*, 2009), and the decrease in *K*^trans^ in solid tumours is concerned with the anti-tumour effect (Morgan *et al*, 2003; Marzola *et al*, 2004; Nakamura *et al*, 2006; Flaherty *et al*, 2008). In this study, *K*^trans^, IAUGC_60_, and *v*_p_ were increased significantly 24 h after the A-83-01 treatment. This increase may be explained by different treatment protocols, different tumour models, and the different signal inhibition between anti-angiogenic agent such as kinase inhibitor and T*β*R-I inhibitor. Similar to A-83-01-treated colon 26 solid tumours, LY364947-treated M109 solid tumours increased IAUGC_60_ at 3 h and recovered fully by 24 h post-injection (Supplementary Figure S2).

It was reported that in a limited situation, anti-angiogenic agents work to deliver more drugs into tumours through the induction of vascular normalisation (Jain, 2001). Untreated colon 26 tumours showed low permeability, in spite of the absence of pericytes or the leaky vessel state (Figure 2B, 3B). The increased *K*^trans^ and IAUGC_60_ values were related to the increased number of growth state cells around the tumour vessel, and were correlated to the decreased *apparent diffusion coefficient value*. Because of no significant difference in tumour cell shape after treatment (Figure 3A), intra-cellular volume did not change. Decreased *apparent diffusion coefficient*, therefore, reflected a decrease in extra-cellular fluid, suggesting that the recovery of delivery may be related to vessel normalisation.

Furthermore, similar to negative correlation between tumour interstitial fluid pressure and permeability of tumour (Haider *et al*, 2007), IAUGC, *K*^trans^, and *v*_p_ showed a moderate negative correlation to *apparent diffusion coefficient*, suggesting that these parameters may be providing similar information. As *apparent diffusion coefficient* is acquired in clinic widely to detect and diagnose a tumour, it could apply conveniently to examine the permeability of tumour in patients.

Although there is room for improvement, DCE-MRI using liposomal contrast agents such as Gd-L could be an important method to anticipate the extravasation of the liposomal anti-cancer drug during T*β*R-I inhibitor-combined therapy.

In summary, we found that DCE-MRI parameters, *K*^trans^, IAUGC_60_, and *v*_p_ were positively related to tumour vasculature by the treatment of A-83-01. Thus, T*β*R-I inhibitor has the potential to enhance the delivery of liposomal anti-cancer drugs and contrast agents. DCE-MRI forms a capable tool to determine the administration schedule of combination therapy with T*β*R-I inhibitor by *K*^trans^ and *v*_p_ quantitation.
