# Supplementary material for: Increase in tumour permeability following TGF-β type I receptor-inhibitor treatment observed by dynamic contrast-enhanced MRI
Source: Br J Cancer. 2009 Nov 3;101(11):1884–90. doi: 10.1038/sj.bjc.6605367 (PMC2788254; doi:10.1038/sj.bjc.6605367)
Supplement: Supplementary Figures S1 and S2 [file 6605367x1.ppt]

## Slide 1
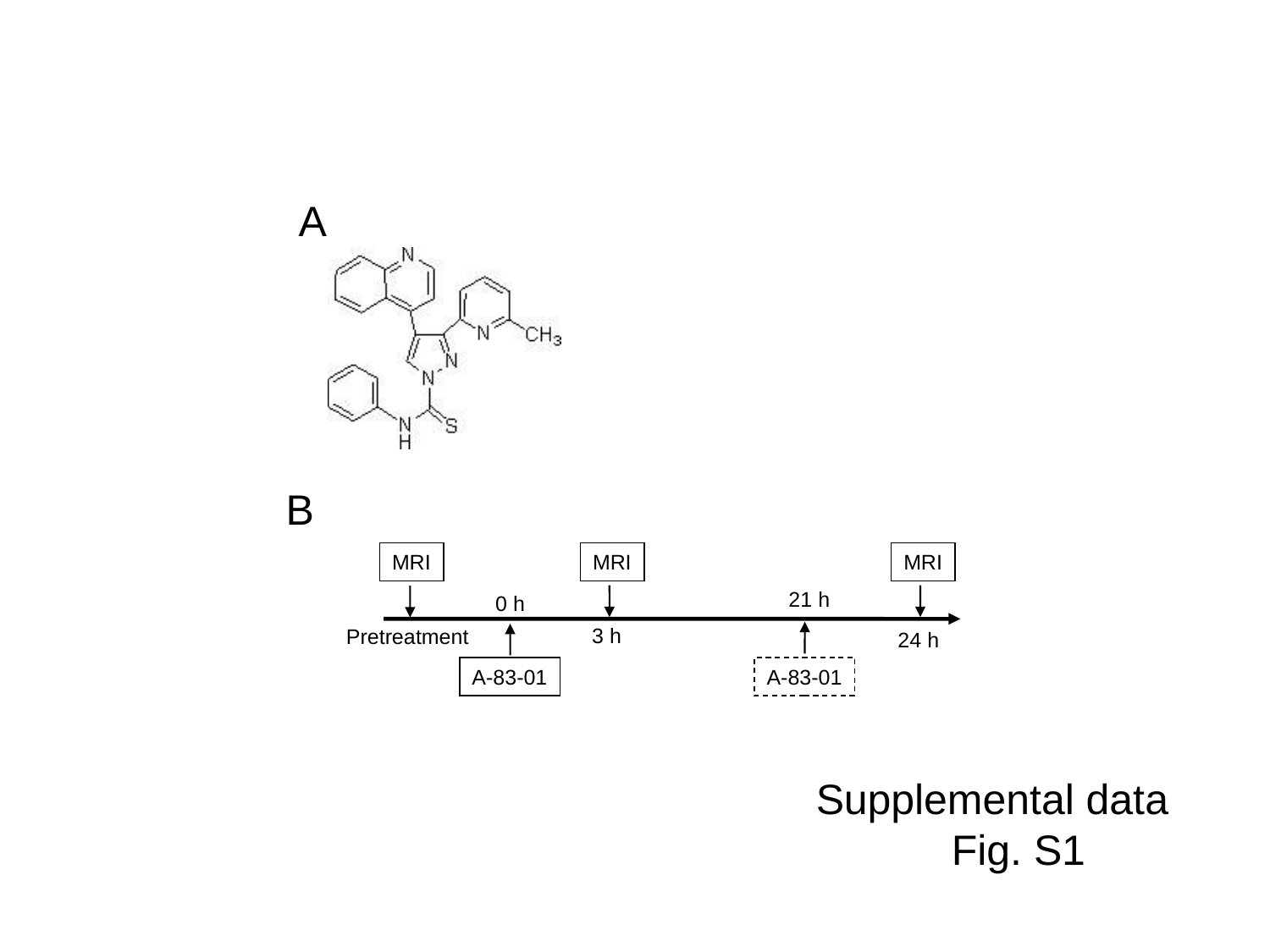

#
A
B
MRI
MRI
MRI
21 h
0 h
3 h
Pretreatment
24 h
A-83-01
A-83-01
Supplemental data　
Fig. S1

## Slide 2
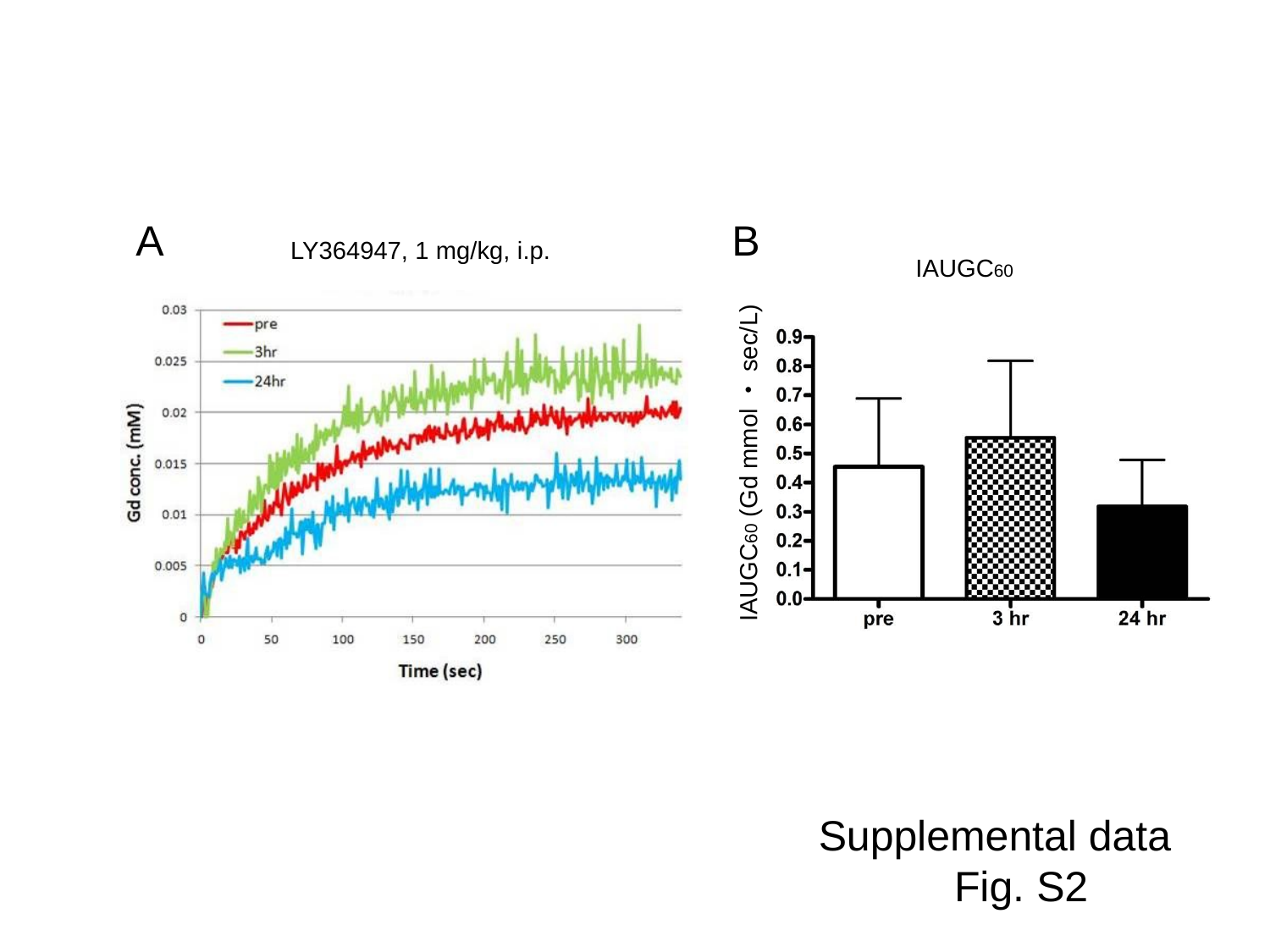

A
B
LY364947, 1 mg/kg, i.p.
IAUGC60
IAUGC60 (Gd mmol・sec/L)
# Supplemental data　 Fig. S2
