# Supplementary material for: Increase in tumour permeability following TGF-β type I receptor-inhibitor treatment observed by dynamic contrast-enhanced MRI
Source: Br J Cancer. 2009 Nov 3;101(11):1884–90. doi: 10.1038/sj.bjc.6605367 (PMC2788254; doi:10.1038/sj.bjc.6605367)
Supplement: Supplementary Figures Legends [file 6605367x2.doc]

Fig. S1 Chemical structure of A-83-01 (A) and protocol of the experiment (B). Gd-DTPA was used in mice with DCE-MRI three times, pretreatment, at 3 h and 24 h. Gd-L was used at pretreatment and at 24 h. Single-treated mice were intraperitoneally injected with A-83-01 at 0 h, and repeat-treated mice were injected at 0 h and 21 h.

Fig. S2 Mean Gd uptake curves from ROIs over whole tumours pretreatment and at different time points posttreatment with intraperitoneal LY364947 injection with Gd-DTPA (A) and IAUGC_60_ (B) in M109 murine lung tumour. Data points indicate mean±SD (N= 3).
